# Intraoperative Radiation for Pancreatic Cancer

**DOI:** 10.1016/j.semradonc.2025.07.008

**Published:** 2025-10

**Authors:** Ahmed Elguindy, Dukagjin Blakaj, John Grecula, Eric D. Miller

**Affiliations:** * Department of Radiation Oncology, The Ohio State University Comprehensive Cancer Center, Columbus, OH.; † Department of Radiation Oncology, Assistant lecturer, El-Demerdash Hospitals, Ain Shams University, Cairo, Egypt.

## Abstract

Pancreatic cancer (PDAC) remains a challenging disease to treat with a poor prognosis. Management of PDAC has evolved over the last several decades with the development of more effective systemic therapy making local control of the primary tumor paramount in patients with both resectable and unresectable disease. Local recurrence after resection or progression of an unresectable tumor are significant causes of morbidity and potential mortality for patients with PDAC. Emerging data in PDAC suggest that improved local control and favorable survival can be achieved with radiation dose escalation. However, adjacent radiosensitive organs limit the ability to deliver higher doses of radiation therapy to patients. Intraoperative radiation therapy provides an ideal way to deliver large doses of radiation directly to the tumor or tumor bed while minimizing the radiation dose to adjacent normal organs. The purpose of this review is to provide an overview of the current literature demonstrating the utility of intraoperative radiation therapy in patients with resectable and unresectable PDAC.

## Introduction

Pancreatic cancer (PDAC) continues to remain one of the deadliest cancers with an estimated 67,440 new cases and 51,980 deaths in the United States in 2022.^1^ Globally, the incidence and mortality of PDAC is rising and is predicted to be the second leading cause of cancer-related death by 2030.^2^ Surgery remains the only curative treatment modality for patients with localized disease, however, only 15%−20% of patients are candidates for surgical resection at diagnosis.^3^ For patients who present with involvement of adjacent vasculature with either borderline resectable or locally advanced disease, neoadjuvant therapy in the form of systemic therapy can be used to both address potential occult micrometastatic disease and to downstage the tumor, making unresectable tumors potentially. Radiation therapy (RT) can also be used as part of neoadjuvant therapy to facilitate a margin negative resection (R0) or to maximize local control (LC) in patients with unresectable disease.^4,5^ Despite the best available therapies, outcomes in pancreatic cancer remain poor with the lowest survival rate of all major organ cancers.^1^ Resection rates for patients with borderline or locally advanced disease remain suboptimal.^6,7^ For patients who are able to undergo resection, rates of local recurrence remain high with reports of up to 53% in some prospective studies.^8–10^ For patients who have unresectable disease, the prognosis is quite poor and marginally better than patients with metastatic disease.^11^ Thus, there is a desperate need for more effective systemic and local therapies for PDAC.

**Figure 1:**
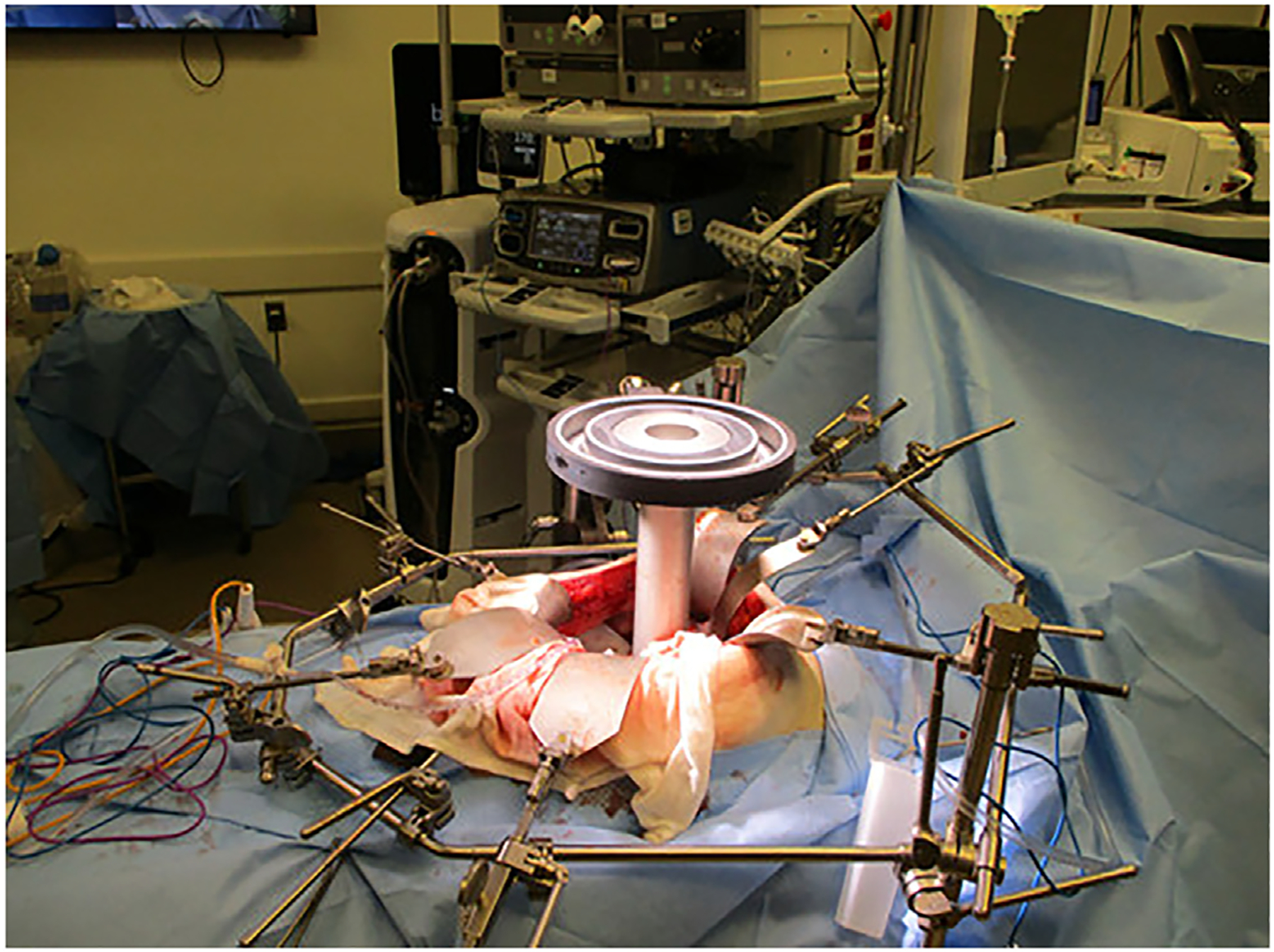
Intraoperative radiation therapy using electrons in a patient with unresectable adenocarcinoma of the pancreatic head.

Emerging evidence suggests that higher radiation doses (biologically effective doses of approximately 100 Gy or higher) can provide durable locoregional control and favorable survival rates in patients with locally advanced PDAC.^12,13^ However, the main limitation to radiation dose escalation for patients with PDAC is the tolerance of nearby luminal gastrointestinal organs. Intraoperative delivery of RT has the advantage of being able to directly visualize the targeted treatment area and adjacent organs at risk with the potential to shield or move these organs from the treatment field.^14^ Thus, for cases where surgical margins are close or involved, IORT can be used to deliver a focal RT boost to the tumor or postoperative bed. IORT delivery can be accomplished using various modalities including electrons, high-dose rate (HDR) brachytherapy, and kilovoltage (KV) X-rays. Modern IORT with electrons involves placement of an applicator over the area of planned treatment followed by docking of a mobile linear accelerator and treatment delivery (Fig. 1). After docking, treatment generally takes mere minutes, and this technique has the advantage of greater depth of penetration and dose homogeneity in comparison to the other available modalities.^14^ Electrons also have the advantage of rapid fall off which aids in sparing organs deeper than the target.^15^ However, there are limitations to the field size that can be delivered and some body sites (for example, low pelvis) are challenging to treat using this technique. Most of the studies using IORT for PDAC in the literature have used electrons as treatment. HDR brachytherapy involves placing flexible applicators on the treatment area which are then connected to an HDR after loader for treatment. HDR IORT has the advantage of being able to treat uneven surfaces, large areas, and difficult to reach places, but has a limited depth of penetration and significantly longer treatment times than with electrons.^14^ KV IORT has a very limited depth of penetration and is primarily used in the treatment of breast cancer.

Modern approaches to IORT were initially investigated in the 1960s in Japan for use in patients with locally advanced abdominal and pelvic tumors, such as gastrointestinal cancers and retroperitoneal sarcoma.^16^ Given the challenges of obtaining a margin negative resection in patients with PDAC, IORT has been of interest in this malignancy for decades. The purpose of this review is to provide an overview of the role of IORT in PDAC including the potential utility of IORT in patients with either unresectable or resectable disease.

## Unresectable Pancreatic Cancer

Surgical removal of the primary tumor remains the best chance for cure in patients with PDAC. For those with tumors that are not amenable to resection, LC of the primary tumor is essential in preventing further morbidity and potential mortality.^17^ Autopsy series have suggested that locally destructive disease as the direct cause of death in up to 30% of patients with pancreatic cancer.^18^ IORT allows for potential radiation dose escalation at the time of surgery for patients deemed to have unresectable disease.

Nishimura et al. reported one of the first retrospective series on the role of IORT in unresectable PDAC.^19^ Thirty-three (47%) out of 70 patients received IORT with doses ranging from 20 to 40 Gy. Excellent palliation of pain was reported in 50% of patients with that complaint. In Stage IV patients, a significant survival difference was observed between those patients who received IORT (4.6 months) vs the control group (2.5 months), *P* < 0.05.

Following that initial publication, other single institution series were reported. Roldan et al. from Mayo clinic reported results of 159 patients with unresectable PDAC who received postoperative external beam radiation therapy (EBRT) (range: 40–60 Gy) with 37 (23%) receiving an additional IORT boost.^20^ Adjuvant chemotherapy with 5-fluorouracil (5-FU) was administered in 132 patients (88% of EBRT alone, 65% of EBRT+IORT). LC at 1 year and 2 years was 82% (EBRT+IORT) vs 48% (EBRT alone) and 66% (EBRT +IORT) vs 20% (EBRT alone), respectively (*P* < 0.005). However, due to the high incidence of hematogenous and/or peritoneal spread in both groups (abdominal failure in 56% (EBRT alone) and 54% (EBRT+IORT) of patients), the LC difference did not result in a meaningful survival difference (median OS 12.6 months (EBRT alone) vs 13.4 months (EBRT+IORT), *P* = 0.25). These initial results demonstrated the utility of IORT in patients with unresectable PDAC, but also revealed the lack of effective systemic therapy at that time which ultimately drove survival.

Early promising data with IORT for PDAC led to a study in the cooperative group setting with RTOG 8505.^21^ This study included 51 patients with locally unresected nonmetastatic PDAC and investigated IORT plus adjuvant chemoradiation. Patients were treated with a combination of 20 Gy of IORT and postoperative EBRT 50.4 Gy in combination with 5-FU. Median survival was 9 months with an 18-month actuarial survival rate of 9%. LC was not evaluated, and major postoperative complications occurred in 12% of patients.

Mohiuddin et al. reported on 49 patients with localized unresectable PDAC treated with IORT (15–20 Gy) and perioperative chemotherapy (5-FU) followed by 5-FU-based chemoradiation (40–55 Gy) and then maintenance chemotherapy.^22^ The median survival time was 16 months, with a 2-year survival rate of 22% and a 4-year survival rate of 7%. Freedom from local progression was achieved in 71% of patients. Early grade 3/4 toxicity was observed in 14% of patients and late grade 3/4 toxicity was reported in 19% of patients, primarily gastrointestinal. It should be highlighted that unlike in RTOG 8505, chemotherapy was administered before and after IORT, combined with EBRT and subsequently delivered as maintenance therapy.

The Japanese Radiation Oncology Study Group reported their results on 144 patients with unresectable PDAC treated with IORT, with or without EBRT, and with or without chemotherapy.^23^ The majority (78.5%) were treated with IORT + EBRT and 79.2% also received systemic therapy, primarily gemcitabine. The median doses of IORT and EBRT were 25 Gy and 45 Gy, respectively. For all patients, the 2-year LC was 44.6%. As expected, patients who received IORT with EBRT had more favorable LC (2-year LC, 50.9%) than those treated with IORT alone (2-year LC, 50.9% vs 17.5%, *P* = 0.0004). Including all patients, the 2-year OS rate was 14.7% and the median OS was 10.5 months. Receipt of chemotherapy was associated with improved OS. Late grade 3 gastrointestinal toxicity was observed in 1.4% of patients.

Jingu et al. reported their experience at Tohoku University Hospital in Japan of 192 patients with localized PDAC treated with IORT, including patients with both resectable and unresectable disease in their analysis.^24^ Of the 192 patients, 48 underwent R0 resection, 35 underwent an R1 resection while 109 patients only received a biopsy or palliative resection. Adjuvant EBRT was delivered in 55 patients and 124 received adjuvant chemotherapy. The median dose of IORT was 25 Gy while the median dose of EBRT was 40 Gy. For all patients, the 2-year LC and OS rates were 71.0% and 16.9%, respectively. Late grade 4/5 gastrointestinal toxicity was observed in 2.1% of patients. As expected, degree of resection (R0–1 vs R2, hazard ratio (HR) = 1.97, *P* = 0.001) and delivery of adjuvant chemotherapy (HR=1.54, *P* = 0.028) were associated with improved OS.

In another large retrospective study, Chen et al. reported on 247 patients with localized locally advanced PDAC who underwent IORT at China National Cancer Center.^25^ A minority of patients received additional treatment. Adjuvant chemoradiation was delivered in 49 patients, adjuvant EBRT alone in 12 patients, and adjuvant chemotherapy only in 37 patients. The median IORT dose was 15 Gy with a range of 10–20 Gy. In addition to IORT, 101 patients also underwent intraoperative interstitial chemotherapy with sustained-release 5-FU implants. The 1-, 2-, and 3-year actuarial OS rates were 40%, 14%, and 7.2%, respectively, with a median OS of 9.0 months. The 1-, 2-, and 3-year local progression-free survival rates were 51.3%, 40.1%, and 34.6%, respectively with a median time to local progression of 8.3 months. On multivariate analysis, smaller IORT applicator size (<6 cm) (HR = 0.67; 95% CI, 0.47–0.97), and postoperative chemoradiation followed by chemotherapy (HR = 0.11; 95% CI, 0.04–0.25) were significantly associated with improved OS. The authors also reported that in 117 patients who complained of pain prior to treatment, nearly 95% had some improvement in their pain after IORT including 74 with complete relief and 37 with partial relief. Postoperative complications were observed in 15.4% of all patients treated with IORT.

Long term results from Massachusetts General Hospital (MGH) were reported by Cai et al.^26^ This study included 194 patients with unresectable PDAC treated with IORT. Nearly all patients (97%) received neoadjuvant chemoradiation, although some patients received split course EBRT before and after IORT. The IORT dose delivered ranged between 10 and 25 Gy with a median of 20 Gy. About 1/3 of patients received induction chemotherapy prior to IORT and/or maintenance chemotherapy after IORT. OS rates at 1-, 2-, and 3-years were 49%, 16%, and 6%, respectively. The median OS was 12.0 months. The 2-year local progression-free survival and distant metastasis-free survival rates were 41% and 28%, respectively. Similar to other studies, multivariate analysis showed that smaller field size (IORT applicator diameter <8 cm) and administration of chemotherapy were associated with improved OS. Harrison et al. presented updated results from MGH in a more modern treatment era which included FOLFIRINOX.^27^ In this retrospective study, they reported on 158 patients with borderline resectable or locally advanced PDAC treated with IORT. The majority of patients received neoadjuvant FOLFIRINOX (83%) and 95% of patients then received neoadjuvant chemoradiation with 5% receiving either stereotactic body radiation therapy or proton beam therapy. Of the 158 total patients, 64 had unresectable disease and received IORT with a median dose of 15 Gy. For those patients with unresectable disease who received FOLFIRINOX (46 patients), the median progression-free survival was 14.7 months with an impressive median OS of 23.0 months. Local progression occurred in 15% of patients who received IORT alone. Treatment was well-tolerated with a 30-day hospital readmission rate of 6.3% and an overall complication rate of 20.3%. A summary of select studies which have investigated the use of IORT in patients with unresectable PDAC is provided in Table 1.

## Resectable Pancreatic Cancer

Given the anatomical location and infiltrative nature of PDAC, achieving a margin negative resection is difficult.^28^ IORT is a potential way to escalate the radiation dose to the area of concern while minimizing dose to adjacent organs at risk which are sensitive to radiation therapy. While most of the studies investigating the role of IORT in patients with resectable PDAC are retrospective, there was 1 randomized study performed at the National Cancer Institute.^29^ In this study, 24 patients with resectable PDAC were randomized to receive IORT with a dose of 20 Gy to the resection bed or to standard treatment which included postoperative EBRT for patients with extrapancreatic extension or lymph node positive disease. Of note, the patients included in this study had locally advanced disease that was amenable to resection only after extensive surgery. Thus, toxicity of the study was quite high with 27% perioperative mortality. The median survival of patients treated with IORT was 18 months vs 12 months for patients on the control arm. The local recurrence rate was 100% in the control arm vs 33% in the IORT arm.

The remaining studies in resectable PDAC are single institution retrospective series. Zerbi et al. performed a retrospective analysis of patients at the University of Milan with resectable PDAC comparing 43 patients who had IORT at time of resection vs 47 patients who underwent resection alone.^30^ Patients in the IORT group received intraoperative doses ranging from 12.5 to 20 Gy. Incomplete resections were performed in 39.5% of patients in the IORT group and 34% in the surgery alone group. Adjuvant chemoradiation was delivered in 30.2% of patients in the IORT group and 27.6% of patients in the surgery alone group. While OS was similar between the groups, fewer patients in the IORT group had a local recurrence compared to the surgery alone group (27.0% vs 56.4%, *P* < 0.01). Overall complication rates were similar between the patient groups with 30.1% in the IORT patients vs 25.5% in the surgery only group. In a larger series from the same group, the authors noted a reduction in local failure (60% surgery alone vs 27% with IORT, *P* = 0.04) with the addition of IORT in patients with locally limited disease (stage I/II based on the 1997 AJCC-UICC staging system).^31^ In addition, IORT delivery significantly prolonged time to local failure, time to any failure, and OS when compared to patients who underwent resection alone. For patients with locally advanced disease (stage III/IVA disease), the addition of IORT reduced the local failure rate, but did not impact time to any failure or OS.

The institutional series from Thomas Jefferson included 99 patients with PDAC of whom 37 were treated with IORT and 46 patients who underwent resection without IORT and had adequate follow-up data.^32^ Patients in the IORT group had a higher rate of R1/R2 resections vs the surgery alone group (43% vs 30%). Consequently, adjuvant chemotherapy was delivered more often in patients who received IORT (84% vs 63%) as was adjuvant EBRT (74% vs 66%). IORT doses ranged from 10 to 20 Gy. Outcomes between the IORT and non-IORT groups were similar. The rate of locoregional recurrence was 23% in the IORT group and 39% in the non-IORT group, *P* = 0.20. OS was also similar with a median OS of 19.2 months for the IORT group and 21.0 months for the non-IORT group, *P* = 0.49. Propensity score analysis was also performed, but, again there was no association observed between receipt of IORT and OS or recurrence. Rates of perioperative complications were similar between the groups, 46% with IORT vs 40% in the non-IORT group.

A multi-institutional retrospective series from Europe reported outcomes of 270 patients with PDAC planned for complete resection with IORT.^33^ R0 resections were achieved in 53.4% of patients with R1 resections in 27.4% and R2 resections in 19.2% of patients. Nearly 24% of patients received preoperative EBRT while 40% of patients received additional EBRT after surgery and IORT. Only 12% of patients received concurrent systemic therapy with EBRT. IORT doses ranged from 7.5 to 25 Gy with a median of 15 Gy. The median LC was 15 months with a 5-year LC of 23.3%. Median OS was 19 months with a 5-year OS of 17.7%. Improved LC and OS were observed in patients who received preoperative EBRT compared to patients who received postoperative EBRT alone or only IORT.

Calvo et al. reported on 60 patients treated with chemoradiation either before surgery (*n* = 19) or after surgery (*n* = 41) with (*n* = 29) or without an IORT boost.^34^ Adjuvant chemotherapy was delivered in 62% of patients. An R1 resection was performed in 41% of the IORT group and 45% of the non-IORT group. IORT doses ranged from 10 to 15 Gy with a median of 15 Gy. Locoregional control and OS at 5 years were 58% and 20%, respectively. On multivariate analysis, margin resection status, higher clinical stage, and not receiving IORT were associated with higher risk for locoregional recurrence. Perioperative complications were similar in the IORT and no IORT groups.

In the more contemporary series from MGH where FOLFIRINOX was used, 86 patients underwent surgical resection with IORT (median dose 10 Gy).^27^ In the patients who received FOLFIRINOX-based neoadjuvant therapy, the median progression-free and OS were 23.5 months and 42.7 months, respectively. Including those who underwent either an R0 or R1 resection, local progression occurred in 12.7% of patients. A follow-up study from the same group investigated the potential for IORT to mitigate positive surgical margins in patients treated with FOLFIRINOX-based neoadjuvant chemotherapy.^35^ The study included 201 patients who received neoadjuvant FOLFIRINOX followed by consolidative radiation with either chemoradiation or proton beam therapy. R1 resection status was present in 18.9% of patients, 16.8% of those who didn’t receive IORT and 21.6% of those who did receive IORT, *P* = 0.468. For all patients, the median disease-free survival and OS were 24 months and 47 months, respectively. In patients who did not receive IORT, R1 resection was associated with worse disease-free and OS. However, in those who received IORT, there was no difference in disease-free or OS in patients who underwent an R0 vs R1 resection. Further, in multivariate analysis in the IORT group, having an R1 resection was not associated with adverse disease-free or OS.

A more recent phase II trial investigated the role of IORT in 41 patients with resectable PDAC using a 50 kV X-ray source.^36^ The treatment volume included the tumor bed, celiac and superior mesenteric arteries, mesenteric root, portal vein, and other areas considered at risk by the treatment team. The target volume was treated with 10 Gy prescribed at 5 mm depth. All patients also received adjuvant gemcitabine-based chemotherapy. Most patients underwent an R0 resection (75.6%), with 12.2% of patients having a retroperitoneal margin <1 mm, and an additional 12.2% of patients who underwent an R1 resection. The majority of patients also had positive regional lymph nodes (63.4%). While the median follow-up was quite short at 9 months, early results were promising. The 1-year LC and OS rates were 76.4% and 94.1%, respectively. Given the short follow-up of the study, only acute toxicity was reported with postoperative complications observed in 24.4% of patients.

A systematic review and meta-analysis was performed by Jin et al. which included 15 studies and 834 patients (401 resection with IORT, 433 resection without IORT) investigating the role of IORT in resectable PDAC.^37^ In the pooled analysis, the IORT group had favorable OS (median survival rate = 1.20, 95% CI 1.06–1.37, *P* = 0.005) compared to patients who did not receive IORT. In addition, there was a significant reduction in local recurrence in the IORT group compared to the no IORT group (relative risk = 0.70, 95% CI 0.51–0.97, *P* = 0.03). The incidence of postoperative complications and perioperative mortality were similar between the groups. Select studies investigating the role of IORT in resectable PDAC are included in Table 2.

## Future Directions

Treatment of PDAC has certainly evolved since the early applications of IORT in this malignancy. Early on, LC of the primary tumor was generally superseded by distant failure due to the lack of effective systemic therapy agents. As more effective systemic therapies are being developed, the distant failures, while still a problem, have become less prominent shifting the spotlight back to the challenge of LC. When reviewing the available data to date, the most impressive outcomes are those of the modern systemic therapy era where any gains in LC are not overcome by systemic failure. However, to truly determine if IORT adds value in the treatment of PDAC patients, well-designed prospective studies are needed. Many of the current studies are limited by their retrospective data collection, heterogeneous patient populations, and dated treatments. To this end, there are a number of prospective studies that are ongoing or have recently completed accrual with final results pending. The PACER trial (NCT03716531) is a multi-institutional phase II study evaluating the benefit of electron beam IORT in patients with borderline resectable and locally advanced PDAC following induction systemic therapy and neoadjuvant radiation therapy. The primary endpoint of the study is 2-year OS. The PancFORT trial (NCT04090463) is a single arm phase II study conducted at Universita di Verona for patients with borderline resectable PDAC. Patients receive FOLFIRINOX induction systemic therapy then consolidative stereotactic body radiation therapy with IORT delivered at the time of surgery. The primary endpoint of the study is disease-specific survival. Unfortunately, this study is suspended to accrual, so it is uncertain if the study will be completed. A phase I trial (NCT05141513) is underway at Johns Hopkins Hospital in patients with resectable PDAC treated with neoadjuvant systemic therapy and stereotactic body radiation therapy followed by resection investigating the safety of an IORT boost using high dose rate brachytherapy to the triangle volume, an area around the pancreas at high risk for harboring subclinical residual disease.^38^ The study is actively recruiting.

Future studies may also consider the utilization of IORT for its potential immune modulating effects based on preliminary data from Lee et al.^39^ Changes in the immune response of 17 patients who were treated with IORT after resection were compared with patients who underwent resection alone. The investigators found higher levels of cytokines in the PI3K/SMAD pathway in the peritoneal fluid of patients who had received IORT. In addition, the peritoneal fluid of patients treated with IORT inhibited proliferation and invasiveness in pancreatic cancer cell lines. Finally, higher rates of cytotoxic and helper T cells were observed in the patients treated with IORT than in patients without IORT. Similar findings have been reported with the use of IORT in the treatment of breast cancer.^40^ These preliminary but exciting data lay the groundwork for future studies investigating the role of potential IORT to help provide maximum LC, but also potentially help drive the systemic response to influence distant control as well.

## Conclusions

While advances have been made, PDAC remains arguably the most lethal of solid organ malignancies. LC of the primary tumor remains a challenge, both in patients with resectable and unresectable disease. IORT is an ideal method of delivering focused doses of radiation therapy to the areas at highest risk of recurrence while sparing adjacent radiosensitive organs. The available data on the utility of IORT in PDAC shows promising results, but is largely limited to retrospective analyses and heterogeneously treated patients using dated systemic therapy. Modern series show encouraging rates of LC and OS, even in patients with unresectable disease. To fully flesh out the benefit of IORT in PDAC, more prospective studies are needed with several in process. Given current patient outcomes, the optimal treatment regimen for patients with localized PDAC that maximizes both local and systemic control has yet to be defined. Based on the data to date, IORT may play an important role in the multimodality management of PDAC to provide optimal outcomes for patients afflicted with this malignancy.

## Figures and Tables

**Table 1:** Selected Studies of Intraoperative Radiotherapy in Patients with Unresectable Pancreatic Cancer

Study	Patient Number	Chemotherapy (%)	Chemotherapy Type	IORT Dose Range (Gy)	EBRT dose Range (Gy)	Local Control (2-Year)	Median Overall Survival (Months)
Roldan et al.^20^	159 (122 EBRT alone, 37 IORT +EBRT)	88% in EBRT; 65% in EBRT +IORT	5-fluorouracil	20	40–60	20% EBRT vs 66% EBRT+IORT	12.6 vs 13.4
Tepper et al.^21^	51 (IORT+EBRT)	100%	5-fluorouracil	20	50.4	NA	9.0
Mohiuddin et al.^22^	49 (IORT+EBRT)	100%	5-fluorouracil/leucovorin	10–20	40–55	31% local recurrence rate	16.0
Ogawa et al.^23^	144 (113 IORT +EBRT, 31 IORT alone)	79.2%	Primarily gemcitabine/5-fluorouracil	12–35	14–50.8	44.6%	10.5
Chen et al.^25^	247 (90 IORT+EBRT, 157 IORT alone)	19.8% concurrent with EBRT plus adjuvant; 11.7% concurrent with EBRT; 15.0% adjuvant only	Gemcitabine or capecitabine	10–20	36–40	2-year local progression free survival rate of 40.1%	9.0
Cai et al.^26^	194 (97% received pre-IORT EBRT)	34%	5-fluorouracil/leucovorin or gemcitabine	10–25	0–59.4	2-year local progression free survival rate 41%	12.0
Harrison et al.^27^	64 (EBRT+IORT)	100%	Primarily FOLFIRINOX or gemcitabine/capecitabinebased therapy	15 mean dose	50.4–58.8 or SBRT	15% local progression rate	23.0

EBRT: external beam radiation therapy, FOLFIRINOX: 5-fluorouracil, leucovorin, irinotecan, oxaliplatin, Gy: Gray, IORT: intraoperative radiation therapy, OS: overall survival, SBRT: stereotactic body radiation therapy.

**Table 2:** Selected Studies of Intraoperative Radiotherapy in Patients with Resectable Pancreatic Cancer

Study	Patient Number	Chemotherapy (%)	Chemotherapy Type	IORT Dose Range (Gy)	EBRT Dose Range (Gy)	Local Control (2-Year)	Median Overall Survival (months)
Zerbi et al.^30^	43 (postoperative EBRT +/− chemo in 40%)	30.2% concurrent with EBRT; 7.0% adjuvant	5-fluorouracil and epiadryamicin	12.5–20	40–45	Local recurrence in 27%	19.0
Showalter et al.^32^	37 (IORT+EBRT in 23)	84%	Not mentioned	10–20	45–50.4	23% locoregional recurrence	19.2
Valentini et al.^33^	270 (169 IORT +EBRT, 95 IORT alone)	11.8% concurrent chemo with EBRT	Not mentioned	7.5–25	18–61	Median local control of 15 months	19.0
Calvo et al.^34^	29 (IORT+EBRT)	62%	Tegafur	10–15	45–50.4	3-year rate of locoregional control 58% (all patients)	3-year 24% (all patients)
Harrison et al.^27^	86 (EBRT+IORT)	100%	Primarily FOLFIRINOX or gemcitabine/capecitabinebased therapy	10 mean dose	50.4–58.8 or SBRT	12.7% local recurrence rate	46.7
Sekigami et al.^35^	201 (EBRT+IORT)	100%	FOLFIRINOX	10	25–50.4	N/A	47.0
Cho et al.^36^	41 (IORT alone)	95%	Gemcitabine-based chemotherapy	10	N/A	1-year local control 76.4%	1-year 94.1%

Abbreviations: EBRT, external beam radiation therapy, FOLFIRINOX, 5-fluorouracil, leucovorin, irinotecan, oxaliplatin, Gy, Gray, IORT, intraoperative radiation therapy, N/A, not applicable, OS, overall survival, SBRT, stereotactic body radiation therapy.

## Data Availability

No data was used for the research described in the article.
